# Development and characterization of acidic-pH-tolerant mutants of *Zymomonas mobilis* through adaptation and next-generation sequencing-based genome resequencing and RNA-Seq

**DOI:** 10.1186/s13068-020-01781-1

**Published:** 2020-08-13

**Authors:** Qing Yang, Yongfu Yang, Ying Tang, Xia Wang, Yunhao Chen, Wei Shen, Yangyang Zhan, Junjie Gao, Bo Wu, Mingxiong He, Shouwen Chen, Shihui Yang

**Affiliations:** 1 State Key Laboratory of Biocatalysis and Enzyme Engineering, Environmental Microbial Technology Center of Hubei Province and School of Life Sciences, Hubei University, Wuhan, 430062 China; 2 Key Laboratory of Development and Application of Rural Renewable Energy, Biomass Energy Technology Research Centre, Biogas Institute of Ministry of Agriculture, South Renmin Road, Chengdu, 610041 China

**Keywords:** *Zymomonas mobilis*, Adaptive laboratory evolution (ALE), Acidic pH tolerance, Next-generation sequencing (NGS), RNA-Seq, Whole-genome resequencing (WGR)

## Abstract

**Background:**

Acid pretreatment is a common strategy used to break down the hemicellulose component of the lignocellulosic biomass to release pentoses, and a subsequent enzymatic hydrolysis step is usually applied to release hexoses from the cellulose. The hydrolysate after pretreatment and enzymatic hydrolysis containing both hexoses and pentoses can then be used as substrates for biochemical production. However, the acid-pretreated liquor can also be directly used as the substrate for microbial fermentation, which has an acidic pH and contains inhibitory compounds generated during pretreatment. Although the natural ethanologenic bacterium *Zymomonas mobilis* can grow in a broad range of pH 3.5 ~ 7.5, cell growth and ethanol fermentation are still affected under acidic-pH conditions below pH 4.0.

**Results:**

In this study, adaptive laboratory evolution (ALE) strategy was applied to adapt *Z. mobilis* under acidic-pH conditions. Two mutant strains named 3.6M and 3.5M with enhanced acidic pH tolerance were selected and confirmed, of which 3.5M grew better than ZM4 but worse than 3.6M in acidic-pH conditions that is served as a reference strain between 3.6M and ZM4 to help unravel the acidic-pH tolerance mechanism. Mutant strains 3.5M and 3.6M exhibited 50 ~ 130% enhancement on growth rate, 4 ~ 9 h reduction on fermentation time to consume glucose, and 20 ~ 63% improvement on ethanol productivity than wild-type ZM4 at pH 3.8. Next-generation sequencing (NGS)-based whole-genome resequencing (WGR) and RNA-Seq technologies were applied to unravel the acidic-pH tolerance mechanism of mutant strains. WGR result indicated that compared to wild-type ZM4, 3.5M and 3.6M have seven and five single nucleotide polymorphisms (SNPs), respectively, among which four are shared in common. Additionally, RNA-Seq result showed that the upregulation of genes involved in glycolysis and the downregulation of flagellar and mobility related genes would help generate and redistribute cellular energy to resist acidic pH while keeping normal biological processes in *Z. mobilis*. Moreover, genes involved in RND efflux pump, ATP-binding cassette (ABC) transporter, proton consumption, and alkaline metabolite production were significantly upregulated in mutants under the acidic-pH condition compared with ZM4, which could help maintain the pH homeostasis in mutant strains for acidic-pH resistance. Furthermore, our results demonstrated that in mutant 3.6M, genes encoding F_1_F_0_ ATPase to pump excess protons out of cells were upregulated under pH 3.8 compared to pH 6.2. This difference might help mutant 3.6M manage acidic conditions better than ZM4 and 3.5M. A few gene targets were then selected for genetics study to explore their role in acidic pH tolerance, and our results demonstrated that the expression of two operons in the shuttle plasmids, *ZMO0956–ZMO0958* encoding cytochrome bc1 complex and *ZMO1428–ZMO1432* encoding RND efflux pump, could help *Z. mobilis* tolerate acidic-pH conditions.

**Conclusion:**

An acidic-pH-tolerant mutant 3.6M obtained through this study can be used for commercial bioethanol production under acidic fermentation conditions. In addition, the molecular mechanism of acidic pH tolerance of *Z. mobilis* was further proposed, which can facilitate future research on rational design of synthetic microorganisms with enhanced tolerance against acidic-pH conditions. Moreover, the strategy developed in this study combining approaches of ALE, genome resequencing, RNA-Seq, and classical genetics study for mutant evolution and characterization can be applied in other industrial microorganisms.

## Background

With the global climate change caused by burning fossil fuels and the growing demand for energy [1, 2], sustainable bioenergy has drawn great attentions [3]. Currently, bioethanol, an environmental-friendly renewable liquid biofuel, has been intensively studied as one of the most promising alternatives to fossil fuels [4, 5]. However, bioethanol has been produced primarily from food crops with high content of sugar and starch thus far, which would compete with the food supply and could potentially lead to a global food crisis.

Lignocellulosic materials, derived mainly from agriculture wastes or forestry residues, are the most abundant, low-cost, and promising feedstocks for bioethanol production [6, 7]. However, these biomass resources are naturally recalcitrant, which require deconstruction processes such as size reduction and pretreatment to breakdown the rigid biomass structure to release fermentable sugars for subsequent microbial fermentation [8, 9]. Among different pretreatment methods, acid pretreatment is a prevailing strategy used to break down the hemicellulose component of the lignocellulosic biomass to release pentoses, such as xylose and arabinose, and is usually followed by a subsequent enzymatic hydrolysis step to release hexoses from the cellulose, which can be used as substrates for biochemical production. The acid-pretreated liquor can also be directly used as the substrate for microbial fermentation, which has an acidic pH and contains inhibitory compounds generated during pretreatment and consequently impedes cell growth resulting in reduced ethanol titer and productivity [10, 11].

To minimize the detrimental effect of acidic pH on microbes, acid-pretreated liquor must be neutralized by high-cost processes such as extra chemical addition before microbial fermentation, especially in large industrial scales [12], whereas a natural acidic-pH condition of acid-pretreated liquor provides an opportunity to effectively prevent the potential bacterial contamination and makes the open (non-sterilized) fermentation applicable [13, 14]. It is reported that ethanol production under the non-sterilized condition can save 30 ~ 40% energy consumption and make the process simpler [15]. Hence, it will be ideal to develop more acidic-pH-tolerant strains for ethanol production, which has been developed in species, such as *Escherichia coli* [16, 17] and yeast [12, 18, 19].

*Zymomonas mobilis* is a facultative anaerobic and natural ethanologenic bacterium with desirable industrial biocatalyst characteristics, such as a highly specific rate of sugar uptake, high ethanol yield, no oxygen requirement for cell growth and ethanol fermentation, and a relatively low biomass production during fermentation [20, 21]. In addition, *Z. mobilis* has a generally regarded as safe (GRAS) status [22, 23]. Up until now, many different stress-tolerant strains of *Z. mobilis* have been constructed with enhanced tolerance to acetate [24, 25], furfural [26, 27], and hydrolysate [28, 29]. However, acidic-pH conditions are still a challenge for *Z. mobilis* using lignocellulosic feedstock hydrolyzed by acid as the substrate. For example, *Z. mobilis* NS-7 is an acid-tolerant strain developed by nitrosoguanidine (NTG) mutation and acid medium selection, which can ferment at an acidic pH of 4.5 under non-sterilized condition without being contaminated [15]. *Z. mobilis* GZNS1 is another mutant strain evolved by culturing at pH 4.0 condition that could produce ethanol from acidic kitchen garbage [14]. An increased acid tolerance was also observed in *Z. mobilis* recombinant strain carrying Pbp (proton-buffering peptide, Pbp) from *E. coli* [30].

In addition, some genomic variants relevant to acid tolerance in *Z. mobilis* have been identified. For example, the acetate-tolerant phenotype in AcR mutant may be due to the over-expression of *ZMO0119* encoding Na^+^/H^+^ antiporter resulting from a 1.5-kb deletion in AcR mutant [24, 25]. And single nucleotide variants (SNVs) in genes *ZMO0056* and *ZMO0589*, which encode a glutamine-fructose-6-phosphate aminotransferase and a DNA repair protein RadA, respectively, have been characterized to likely contribute to acid tolerance in mutant stains developed by a multi-round atmospheric and room temperature plasma (mARTP) mutagenesis [31]. Nevertheless, it is still a challenge to develop strains tolerant to acidic pH due to the lack of a comprehensive understanding on functional genomics and molecular regulation responsible for the acid tolerance in *Z. mobilis*.

Adaptive laboratory evolution (ALE) is a powerful tool for strain development with desired stable phenotypes without requiring knowledge of any underlying genetic mechanism [32–34]. It has been successfully applied in model organisms, such as *E. coli* [35, 36] and *Saccharomyces cerevisiae* [37, 38]. After obtaining mutant strains, next-generation sequencing (NGS) based strategies such as whole-genome resequencing (WGR) and RNA sequencing (RNA-Seq) are usually applied to reveal genetic and global gene expression changes [39]. Currently, ALE strategy has been employed for strain improvement in *Z. mobilis*, including inhibitor tolerance [27, 28] and substrate utilization [40–42]. In this study, ALE was performed under acidic-pH conditions to select *Z. mobilis* mutant strains with enhanced acidic pH tolerance. WGR and RNA-Seq technologies were then applied to investigate the genotypic changes associated with acidic pH tolerance revealing the molecular mechanism responsible for the improved acidic pH tolerance in mutant strains.

## Results and discussion

### Development of acidic-pH-tolerant mutants of *Z. mobilis* through adaptive laboratory evolution (ALE)

**Fig. 1:**
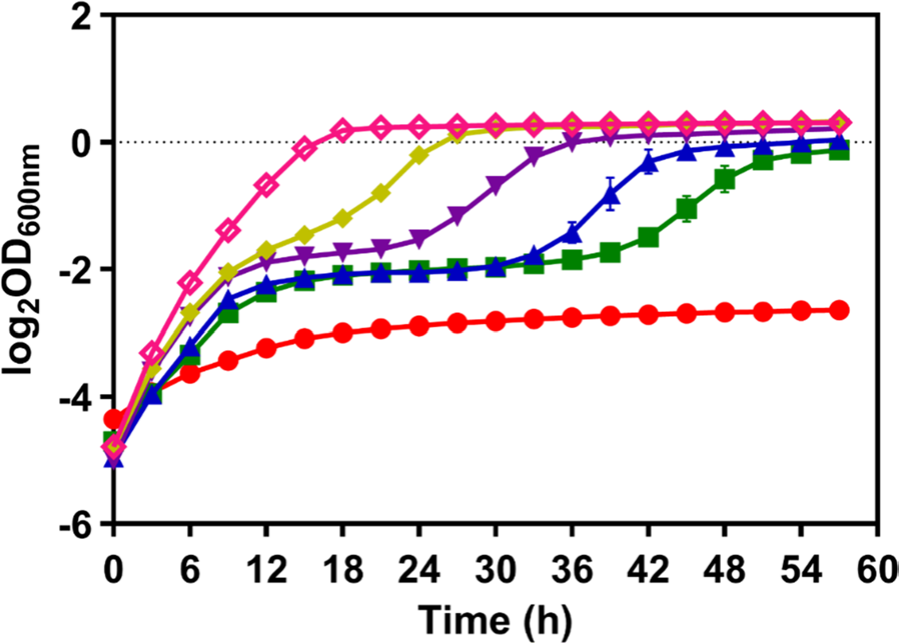
Cell growth of wild-type *Z. mobilis* ZM4 under different pH conditions. ZM4 was cultured in RMG5 using Bioscreen C instrument at a pH range within pH 3.5–4.0. pH 3.5 (red color circle), pH 3.6 (green color square), pH 3.7 (blue color upward triangle), pH 3.8 (purple color downward triangle), pH 3.9 (yellow color diamond), and pH 4.0 (pink color diamond). At least two independent experiments were performed with similar results. Values are the mean of one representative experiment with three technical replicates. Error bars represent standard deviations

*Zymomonas mobilis* was reported to be able to grow within a broad range of pH 3.5 ~ 7.5 [21]. In this study, the growth of *Z. mobilis* in pH range below pH 4.0 was further investigated. The results showed that ZM4 can grow below pH 4.0 (Fig. 1), which is consistent with previous reports [21, 43]. However, when the pH value decreased from 4.0 to 3.5, a longer lag phase was observed accompanied by a lower biomass production (Fig. 1). Cells almost could not grow at pH 3.5 (Fig. 1), which might be ascribed to the damage of cell membrane structure and protein configuration at acidic pH [44]. Therefore, the development of acidic-pH-tolerant strains could directly benefit commercial bioethanol production under acidic fermentation conditions.

**Fig. 2:**
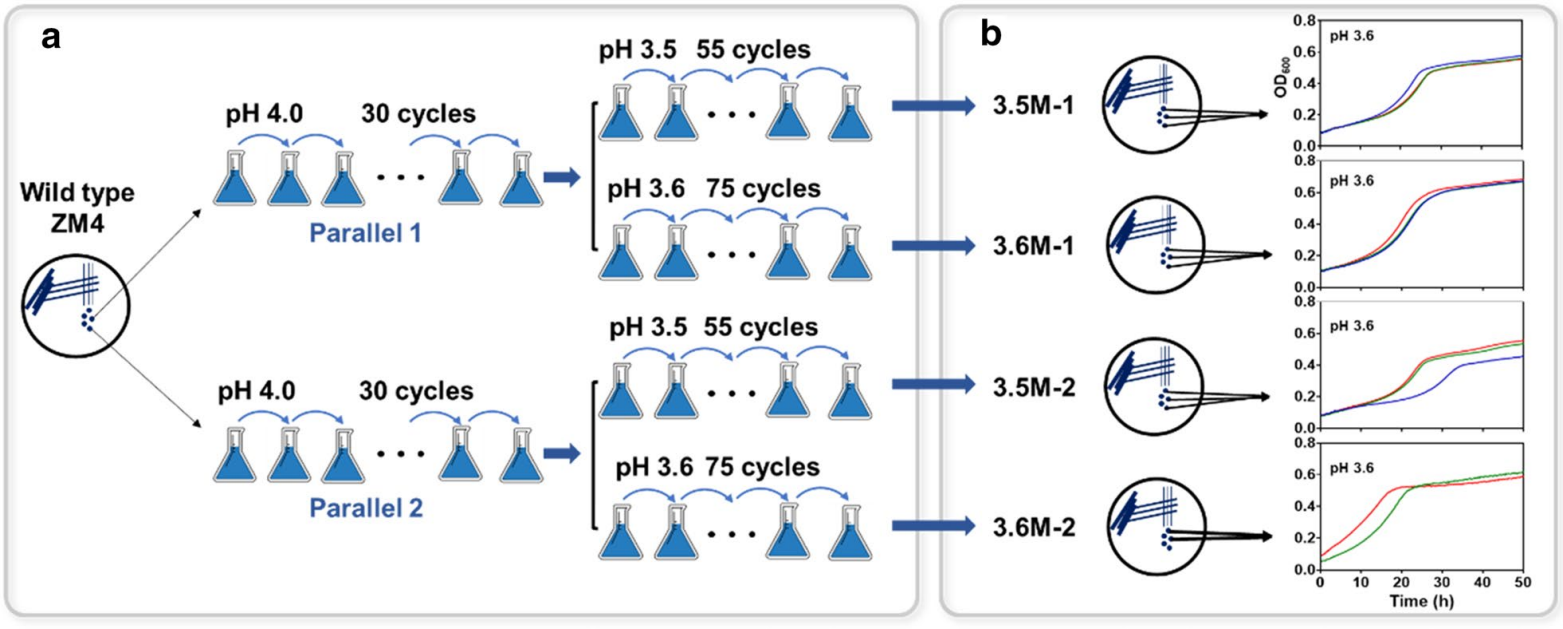
The workflow to obtain acidic-pH resistant mutants of *Z. mobilis* ZM4 through adaptive laboratory evolution (ALE) in RMG2 media (**a**), and the verification and selection of mutants with stable acidic-pH resistance under pH 3.6 culture condition (**b**)

**Fig. 3:**
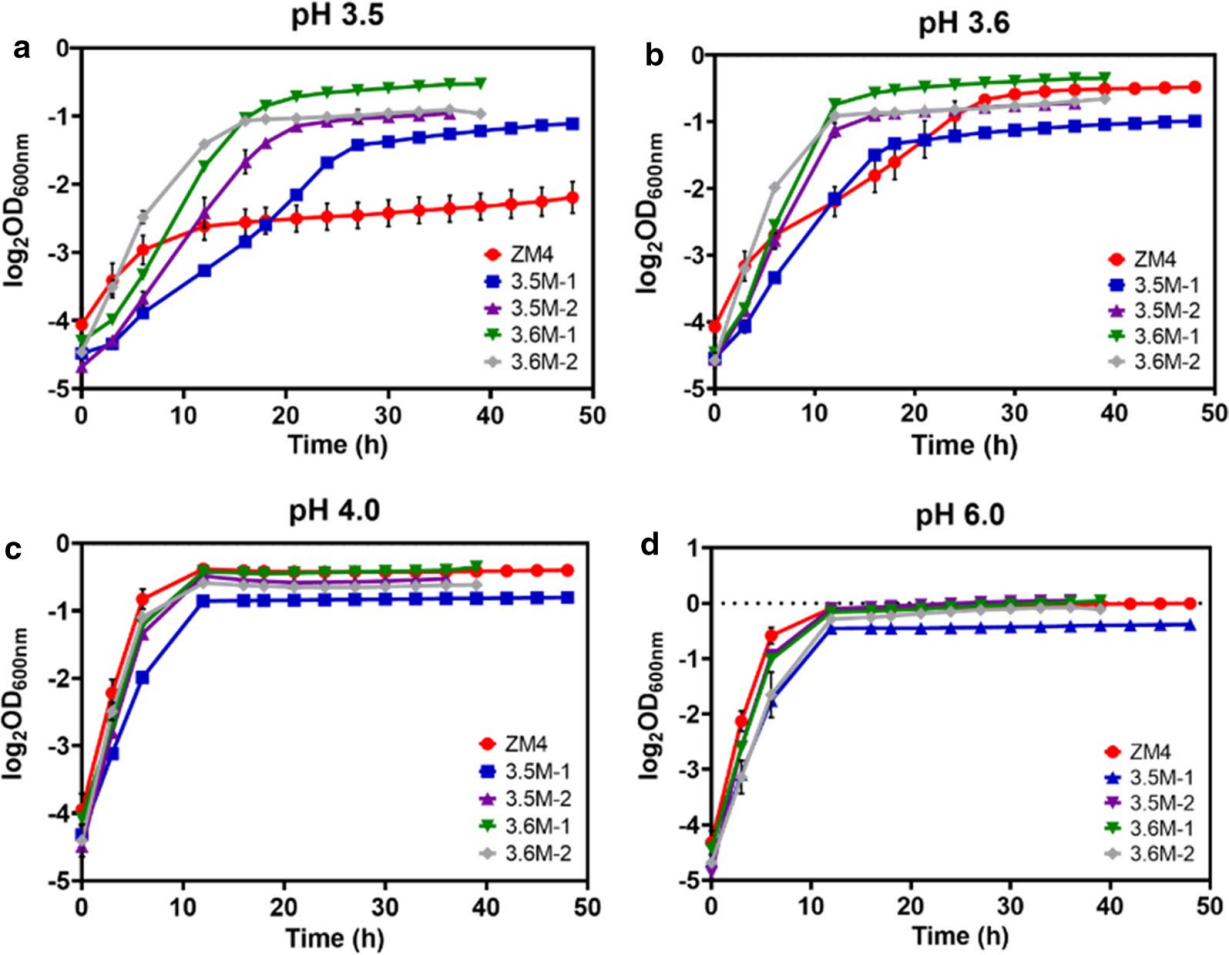
Cell growth of four acidic-pH-tolerant mutants of 3.5M-1, 3.5M-2, 3.6M-1, and 3.6M-2 and wild-type *Z. mobilis* ZM4 under different pH conditions of pH 3.5 (**a**), pH 3.6 (**b**), pH 4.0 (**c**), and pH 6.0 (**d**) in RMG2. OD values at 600 nm were monitored using Bioscreen C instrument. At least two independent experiments were performed with similar results. Values are the mean of one representative experiment with three technical replicates. Error bars represent standard deviations

Subsequently, adaptive laboratory evolution (ALE) was carried out with two parallel experiments in RMG2 medium, which was firstly adapted at pH 4.0 with 30 cultivation cycles and then transferred to pH 3.5 and pH 3.6 for acidic-pH resistance evolution (Fig. 2a). Finally, after 55 and 75 cultivation cycles at pH 3.5 and pH 3.6, respectively, four evolved mutants, named as 3.5M-1, 3.5M-2, 3.6M-1, and 3.6M-2, with enhanced acidic pH tolerance were obtained. The stability of these four adapted mutants was then analyzed at pH 3.6 with three colonies of each as replicates. The results showed that the growth of replicates from 3.5M-1 and 3.6M-1 was more uniformed than those of 3.5M-2 and 3.6M-2 (Fig. 2b). The growth of these four mutants was then further compared with wild-type ZM4 under different pH conditions of pH 3.5, pH 3.6, pH 4.0, and pH 6.0 (Fig. 3).

Under the condition of pH 3.5 (Fig. 3a), all four mutants had higher growth rates and final OD_600_ values than ZM4, among which the mutant 3.6M-1 exhibited the highest OD_600_ value of cell growth, followed by 3.6M-2, 3.5M-2 and 3.5M-1 successively. Under pH 3.6 condition, the mutants also had higher growth rates and shorter times reaching stationary phase than ZM4 (Fig. 3b). The mutants had no obvious disadvantages compared to ZM4 except that 3.5M-1 had a lower final OD_600_ value at both pH 4.0 and pH 6.0 (Fig. 3c, d). These results suggested that although all mutants had enhanced tolerance at acidic-pH conditions, their performance was different. To understand the molecular mechanism of acidic-pH resistance, two mutants 3.5M-1 and 3.6M-1 with growth differences from ZM4 were selected and renamed as 3.5M and 3.6M correspondingly for further studies. 3.6M-1 is the best acidic-pH-tolerant mutant based on its highest final OD_600_ values among all mutants and wild-type ZM4 in acidic-pH conditions. Another acidic-pH-tolerant mutant 3.5M-1 was selected because it grew better than ZM4 at pH 3.5 while having the largest difference from 3.6M-1 in various conditions (Fig. 3).

### Evaluation of cell growth, glucose consumption, and ethanol production of mutant strains 3.5M and 3.6M at acidic and neutral pH conditions

**Fig. 4:**
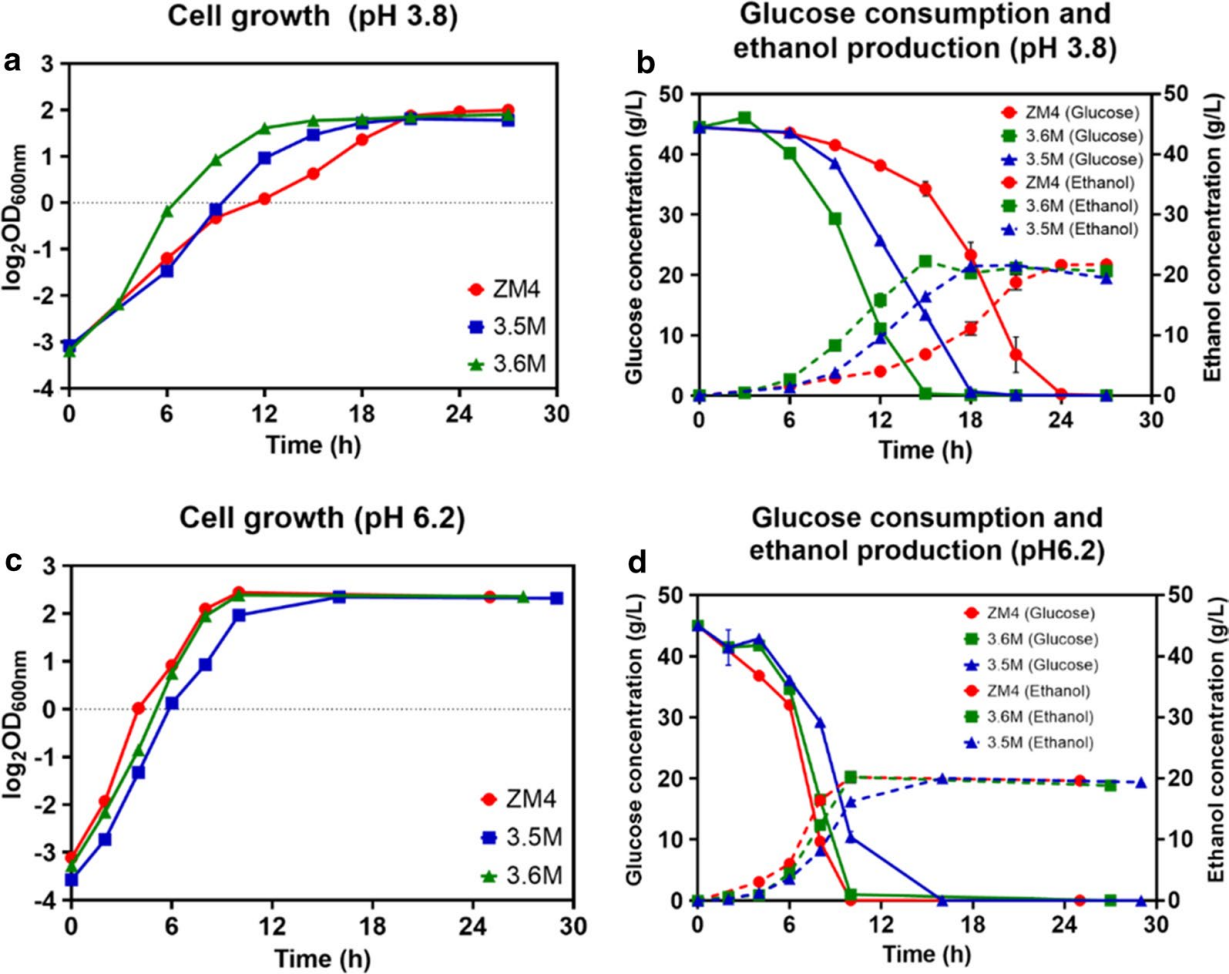
Cell growth, glucose consumption, and ethanol production of *Z. mobilis* mutants 3.5M and 3.6M compared with wild-type ZM4 at pH 3.8 (**a**, **b**) and pH 6.2 (**c**, **d**). At least two independent experiments were performed with similar results. Values are the mean of one representative experiment with three technical replicates. Error bars represent standard deviations

Since the acidic-pH conditions affect cell growth, glucose consumption, and ethanol production, two mutant strains 3.5M and 3.6M were investigated at acidic and neutral pH conditions of pH 3.8 and pH 6.2, respectively. Both mutants 3.5M and 3.6M exhibited better cell growth and faster ethanol production than wild-type ZM4 at acidic pH 3.8 (Fig. 4a, b). The growth rates of 3.5M and 3.6M were 0.23 h^−1^ and 0.35 h^−1^, respectively, while that of ZM4 was only 0.14 h^−1^ (Table 1). Consistent with the fast cell growth and glucose consumption, the fermentation time were reduced significantly from 22 h for ZM4 to 18 h and 13 h for 3.5M and 3.6M, respectively, leading to the increase of ethanol productivity by 21.21% and 64.65% correspondingly (Table 1; Fig. 4a, b).

**Table 1:** Fermentation performance of time to consume all glucose (Time), growth rate, as well as ethanol titer, yield, and productivity of the wild-type *Z. mobilis* ZM4 and mutant strains 3.5M and 3.6M in RMG5 at pH 3.8 and pH 6.2

Condition and Strain	Glucose used (g/L)	Time (h)	Growth rate (h^−1^)	Titer (g/L)	Yield (%)	Productivity (g/L/h)
pH 3.8						
ZM4	44.95 ± 0.12	22	0.14 ± 0.01	21.74 ± 0.43	94.55 ± 1.89	0.99 ± 0.02
3.5M	44.91 ± 0.01	18	0.23 ± 0.01	21.62 ± 0.14	94.13 ± 0.64	1.20 ± 0.01
3.6M	44.98 ± 0.00	13	0.35 ± 0.01	21.22 ± 0.28	92.24 ± 1.22	1.63 ± 0.02
pH 6.2						
ZM4	44.91 ± 0.11	10	0.49 ± 0.007	20.21 ± 1.33	87.99 ± 5.88	2.02 ± 0.13
3.5M	44.97 ± 0.00	12	0.39 ± 0.005	20.05 ± 0.82	87.19 ± 3.56	1.67 ± 0.07
3.6M	44.95 ± 0.00	10	0.49 ± 0.01	20.24 ± 0.36	90.00 ± 1.20	2.02 ± 0.04

At least two independent experiments were performed with similar results. Values are the means and standard deviations of one representative experiment with three technical replicates

Under the neutral pH condition of pH 6.2, cell growth, glucose consumption, and ethanol production of 3.6M were similar to those of ZM4, but were better than those of 3.5M (Table 1; Fig. 4c, d). These results suggested that 3.5M and 3.6M possessed relatively fast glucose consumption and ethanol production at the acidic-pH condition, and 3.6M maintained similar capacities as ZM4 at the neutral pH condition. Thus, 3.6M can be used to replace ZM4 as the biocatalyst for bioethanol production fermenting well in both acidic and neutral pH conditions (Table 1; Fig. 4c, d).

### The underlying mechanism of acidic pH tolerance through NGS-based genome resequencing and RNA-Seq

To illustrate the underlying genetic basis responsible for the enhanced acidic pH tolerance, samples of mutant and wild-type strains cultured at acidic pH 3.8 and neutral pH 6.2 were collected for WGR to determine the genetic changes in 3.5M and 3.6M using the genome of parental strain ZM4 (ATCC 31821) as the Ref. [45]. RNA-Seq was also employed to explore the global transcriptional differences among these strains at acidic and neutral pH conditions.

**Fig. 5:**
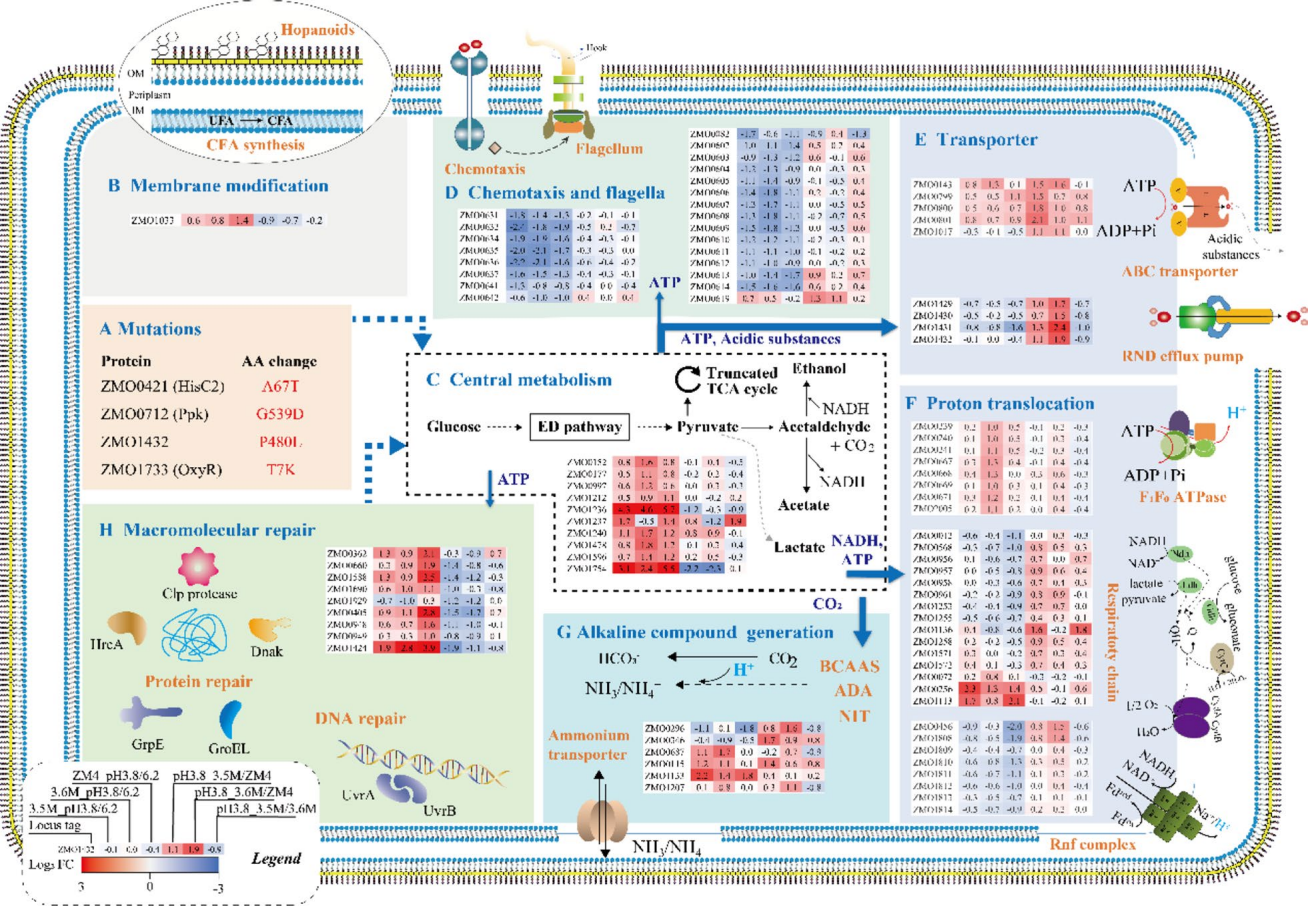
Potential molecular mechanism of acidic-pH-tolerant mutant strains of *Z. mobilis*. Common mutations identified in two mutants (**a**); potential membrane modification (**b**); upregulation of the central metabolism producing enough ATP and reducing power (**c**); downregulation of flagella and chemotaxis reducing energy consumption (**d**); export of acidic substances by transporters (**e**); translocation of excess proton out of cell by F_1_F_o_ ATPase and electronic transport chain related complex (**f**); alkaline compound generation (**g**); downregulation of macromolecular repair system (**h**). The numbers after the gene locus in shadow represent the log_2_-based fold changes. Red indicates upregulated, blue indicates downregulated. BCAAs: branched-chain amino acids, ADA: adenosine deaminase, NIT: nitrilase

The WGR results identified several SNPs in the mutants, including seven SNPs in 3.5M and five SNPs in 3.6M, which are listed in Table 2. Among these mutations, four common SNPs were found in both mutants located at the coding sequence (CDS) region of four genes: *ZMO0421* (A67T), *ZMO0712* (G539D), *ZMO1432* (P480L), and *ZMO1733* (T7K), respectively (Fig. 5a). These common mutations may contribute to the enhanced acidic pH tolerance of mutant strains, while other unique mutations that are not shared by these strains may contribute to the unique phenotypic differences of these strains.

**Table 2:** Single-nucleotide polymorphisms (SNPs) in mutant strains 3.5M and 3.6M compared to wild-type ZM4

Locus	SNP	AA change	3.5M	3.6M	Gene	Product
424761	C/T	A67T	99.76	98.86	*ZMO0421 (hisC2)*	Histidinol-phosphate aminotransferase
711194	G/A	G539D	99.24	99.73	*ZMO0712* (*ppk*)	Polyphosphate kinase
1449594	G/A	P480L	100	99.72	*ZMO1432*	Efflux pump membrane component
1779278	C/A	T7K	100	99.71	*ZMO1733* (*oxyR*)	Transcriptional regulator OxyR
1306151	C/T	W485*	99.5	–	*ZMO1291*	Peptidase S10 serine carboxypeptidase
1701191	G/A	L77F	99.08	–	*ZMO1651* (*ptsP*)	Signal transduction protein
173653	T/C		100	–	Intergenic region	Between *ZMO0183* and *ZMO0184*
1451222	A/G		–	100	Intergenic region	Between *ZMO1432* and *ZMO1433*

The numbers in the columns of 3.5M and 3.6M represent the frequency (%) of the SNP identified in all genome resequencing reads, and “–” indicates the absence of SNP. AA means amino acid. * indicates stop codon

Additionally, the differentially expressed genes (DEGs) were identified through analysis of variance (ANOVA) using strains and different pH conditions as variables. A total of 781 genes were identified by comparing any two conditions with *P* value < 0.05 (Additional file 1: Table S1). There were 271, 362, and 498 DEGs comparing acidic pH 3.8 with neutral pH 6.2 conditions of 3.5M, 3.6M and wild-type ZM4, respectively (Additional file 2: Fig. S1A). 246, 199 and 144 DEGs were also identified comparing 3.5M with ZM4, 3.6M with ZM4, and 3.5M with 3.6M at acidic pH 3.8 conditions, respectively (Additional file 2: Fig. S1B). The DEGs from comparison of the same strain under different pH conditions or different strains under acidic pH 3.8 conditions were then further analyzed.

#### Association of genes with common changes in mutants with enhanced acidic pH tolerance

A common mutation was found in gene *ZMO0421*, encoding histidinol-phosphate aminotransferase HisC2, which catalyzes the seventh step in the histidine biosynthesis pathway. Previous studies in *Z. mobilis* showed that HisC2 has broad substrate specificity and participates in transamination reactions for tyrosine and aromatic amino acid (phenylalanine) biosynthesis, which is essential in all studied organisms [46]. The A67T mutation in ZMO0421 was located in the amino transfer domain (PF00155, 32-357 aa) catalyzing the transamination reaction, which could likely affect enzymatic activity although detailed experiment is needed in the future.

Another common mutation was found in *ppk* gene (*ZMO0712*), which encodes polyphosphate kinase that transfers the γ-Pi of ATP to form a long chain polyphosphate (polyP) reversibly [47]. Several biological functions have been identified for cellular polyP including buffering capacities for pH homeostasis, DNA damage repair, cell cycle, motility, and biofilm formation [48–50]. Studies in other bacteria showed that polyP was rapidly accumulated by PPK under environmental stresses including acidic conditions [51–53]. Our transcriptomic data indicated that the expression of *ppk* in wild-type ZM4 and mutant strains was upregulated at pH 3.8 compared with pH 6.2 (Additional file 3: Table S2), which is consistent with the conclusion reported above. Considering that the G539D mutation in PPK was located in the C2 domain (PF13090, 503–687), which is highly conserved in the PPK family and essential for the enzymatic activity [53], the mutation in this enzyme may help improve the activity of PPK resulting in the acceleration of polyP production to respond to the toxic acidic conditions.

Additionally, a mutation in gene *ZMO1432* encoding the inner membrane protein component of an RND efflux system containing 12 transmembrane domains [54] was observed in both mutants. The P480L mutation was located at the eleventh transmembrane (TM11) domain, which may play an important role in the process of substrates extrusion from cytoplasm to periplasm by proton motive force (PMF) with the conformational changes of RND system [54]. According to the prediction by TMHMM Server v. 2.0 [55], the transmembrane probability of TM11 domain in the mutant protein was improved from 0.7 to 0.95 (Additional file 4: Fig. S2). Therefore, the P480L mutation in ZMO1432 may increase the stability and rigidity of TM11 and hence indirectly improve the efficiency to resist acidic stress by pumping out toxic substances such as organic acids or anions [56].

Moreover, a mutation (A to G) was also found in the intergenic region between *ZMO1432* and *ZMO1433* in mutant 3.6M (Table 2), which is in the upstream of the promoter region of *ZMO1432* predicted by BPROM [57]. As shown in the RNA-Seq results, the expression of the whole operon encoding an RND efflux system consisted of *ZMO1432*, *ZMO1431*, *ZMO1430* and *ZMO1429* was significantly upregulated at acidic pH 3.8 in two mutant strains compared with ZM4, and 3.6M had the highest expression level among these strains (Additional file 1: Table S1, Additional file 3: Table S2). The mutation in the intergenic region in mutant 3.6M could help upregulate the expression of downstream genes, since the expression of these genes was also upregulated under pH 6.2 in 3.6M compared with ZM4 (Additional file 3: Table S2). Combining these mutations and transcriptomic results, the RND efflux pump may play a crucial role in acidic-pH resistance in mutant strains.

The last common mutation shared in both mutant strains was within *oxyR* gene (*ZMO1733*). OxyR is a LysR family transcriptional regulator consisting of an N-terminal DNA-binding domain (DBD) and a C-terminal regulatory domain (RD), which controls the OxyR regulon consisting of almost 40 genes that can help protect cells from oxidative stress [58]. The T7K mutation in OxyR was in the N-terminal of LysR-type helix–turn–helix (HTH) DNA-binding domain (PS50931, 6-63 aa), which likely changes the binding affinity of HTH with its target DNA sequence due to the amino acid change from threonine with short side chain to lysine with long side chain (Table 2). Our RNA-Seq results showed that several genes involved in reactive oxygen species (ROS) detoxification possibly regulated by OxyR, such as *ZMO0918* (catalase) and *ZMO1060* (superoxide dismutase), were significantly upregulated in all strains, especially in ZM4 at pH 3.8 compared to pH 6.2, while *ZMO1211* (glutathione reductase) was significantly upregulated at pH 3.8 only in wild-type ZM4 (Additional file 3: Table S2). Since acidic pH could induce a secondary oxidative stress and the acid tolerance response overlaps with the oxidative stress response [59, 60], the mutation in *oxyR* could contribute to the acidic pH tolerance in mutant strains.

Since mutant strains with these mutations exhibited advantages under acidic-pH condition compared with wild-type ZM4, these mutations could be crucial for *Z. mobilis* to resist the acidic-pH stress although further investigation is needed to help confirm whether or not and how they are necessary for the acidic-pH resistance phenotype.

#### Upregulation of genes associated with membrane components for enhanced acidic pH tolerance

The modification of the phospholipids in the inner membrane is a strategy to reduce proton permeability, since the lipid composition of cell membrane could be reconfigured at the acidic-pH condition, which will affect proton permeability directly or indirectly [61]. In many bacteria, the resistance to acidic pH is associated with the conversion of unsaturated fatty acids (UFAs) into cyclopropane fatty acids (CFAs) through the addition of a methyl group to the double bond of UFA, which is associated with cyclopropane fatty acid synthase (Cfa). The expression of *cfa* gene is usually upregulated under acidic conditions [62, 63], and a similar upregulation was observed for gene *ZMO1033* encoding Cfa in ZM4 at acidic pH, suggesting that *cfa* gene may be associated with outer membrane modification and acidic pH tolerance (Fig. 5b; Additional file 3: Table S2).

#### Energy generation through increased glycolysis and energy conservation through decreased cell motility for acidic-pH resistance

It is reported that *Streptococcus mutans* altered its metabolism by increasing the glycolytic activity to produce ATP at acidic-pH conditions [64, 65], and ATP utilization was further derived from cell growth for acid tolerance [66]. Although the Entner–Doudoroff (ED) pathway only produces one mole of ATP per single mole of glucose, it is reported that the ED pathway in *Z. mobilis* is nearly twice as thermodynamically favorable as the Embden–Meyerhof–Parnas (EMP) pathway in *E. coli* or *S. cerevisiae* [67]. Our RNA-Seq results demonstrated that four genes involved in the glycolysis pathway, *ZMO1478 (pgl), ZMO1240 (gpmA), ZMO1596 (adhB),* and *ZMO1236 (adhA)*, were significantly upregulated at the acidic pH 3.8 compared to a neutral pH 6.2 in ZM4 and 3.6M. Another three genes involved in glycolysis pathway, *ZMO0997 (eda), ZMO0177 (gap),* and *ZMO0152 (pyk)*, were significantly upregulated at pH 3.8 compared to pH 6.2 only in 3.6M. Since these genes are involved in energy generation and recycling, the upregulation of these genes could help produce more ATP for acidic pH tolerance (Fig. 5c; Additional file 3: Table S2). Correspondingly, the final log_2_OD_600_ values of three strains at pH 3.8 were lower than those at pH 6.2, which was about 1.90 and 2.34, respectively (Fig. 4a, c), indicating that more energy was consumed for acidic-pH resistance instead of cell growth. This energy-demanding process might explain the uncoupling between glycolytic and biosynthetic reactions in *Z. mobilis* [68] with energy generated from glycolysis being diverted for acidic-pH resistance, which is consistent with previous reports that the upregulation of *pyk* enhanced tolerance against acid stress in *S. mutans* [65].

In addition, the expression of *ZMO1754* encoding SsdA that catalyzes the reaction of acetate biosynthesis from acetaldehyde was significantly upregulated in both mutants and especially wild-type ZM4 at pH 3.8 compared to that at pH 6.2, and was significantly downregulated in mutant background compared to ZM4 at pH 3.8 (Fig. 5c; Additional file 3: Table S2). The *ssdA* gene expression was also correlated with acetate production in mutants and wild-type strains (Additional file 5: Fig. S3). These results indicated that more acetate might be produced at an acidic pH than at the neutral pH condition, and acidic-pH-tolerant mutants produced less protonated acetate than wild-type ZM4 in acidic-pH conditions. Therefore, acidic-pH-tolerant mutants could have a less acidified cytoplasmic environment than wild-type ZM4 and could divert NAD^+^ used for acetate production into glycolysis maintaining a low NADH/NAD^+^ ratio, which was reported to be responsive for acetic acid tolerance in *Z. mobilis* [31].

Moreover, a number of genes encoding flagellar structure proteins and chemotaxis-related proteins were significantly downregulated under the acidic pH compared with neutral pH condition in both ZM4 and mutant strains (Fig. 5d; Additional file 3: Table S2), which could also help conserve energy from cell motility for survival in conditions of stress such as acidic-pH and ethanol shock [69].

#### Upregulation of transporter and efflux pump helped maintain pH homeostasis in acidic conditions

The increase of acidic end-products such as acetate in acidic-pH conditions (Additional file 5: Fig. S3) could lead to an acidic intracellular condition [64]. Therefore, it is important for cells to export acidic products to maintain intracellular pH homeostasis. ABC transporters transport a wide spectrum of substrates from small inorganic and organic molecules to larger organic compounds, and have been confirmed to contribute to acetic acid tolerance as an efflux pump of acetic acid [70]. Our results demonstrated that five genes encoding ABC transporters (*ZMO0143, ZMO1017, ZMO0799–ZMO0801*) were significantly upregulated in both mutant strains compared with ZM4 at pH 3.8 (Fig. 5e; Additional file 3: Table S2). Moreover, RND efflux pump is well-known for transporting various compounds including cationic dyes, antibiotics, detergents, and even simple organic solvents with the proton antiport [56, 71, 72]. Our results indicated that an RND efflux pump encoded by *ZMO1429–ZMO1432* was also significantly upregulated at acidic pH in both mutant strains compared with ZM4 (Fig. 5e; Additional file 3: Table S2). The upregulation of ABC transporters and efflux pumps may suggest an enhanced capability of mutant strains to maintain cytoplasmic pH homeostasis under acidic-pH conditions.

In addition, pumping H^+^ out of the cytoplasm is another efficient way to maintain pH homeostasis [73]. F_1_F_o_ ATP synthase (F_1_F_o_ ATPase) can utilize the proton gradient for ATP synthesis; it can also reverse and hydrolyze ATP to pump H^+^ out to maintain intracellular pH homeostasis [74, 75]. For example, genes encoding F_1_F_o_ ATPase in *S. mutans* were upregulated at acidic pH to help resist acid stress [76]. Another study indicated that when respiration was impeded, F_1_F_o_ ATPase hydrolyzed ATP to pump protons and contributed to the intracellular neutral condition maintaining the essential mitochondrial membrane potential [77]. Our results demonstrated that 7 genes encoding F_1_F_o_ ATP synthase (*ZMO0239*, *ZMO0240*, *ZMO0241*, *ZMO0667*, *ZMO0668*, *ZMO0669*, *ZMO0671*) and another gene encoding F_1_F_o_ ATP synthase assembly protein (*ZMO2005*) were significantly upregulated at pH 3.8 compared to pH 6.2 for the mutant strain 3.6M (Fig. 5f; Additional file 3: Table S2). Since the cellular respiration process was uncoupled with cell growth in *Z. mobilis* [78], and the ATP generation was majorly from glycolysis whose activity was increased as discussed above, the upregulation of F_1_F_o_ ATPase genes may possibly help pump H^+^ out from the cytoplasm through consuming ATP.

Furthermore, proton translocation was suggested to result in an alkalization of the intracellular medium in *Z. mobilis* at pH 6.5 during the respiration by transferring the H^+^ out of cytoplasm [79]. Two genes related to the respiration chain for transferring electrons to oxygen, *ZMO0012* and *ZMO0568,* were downregulated significantly; and six other genes*, ZMO0956–ZMO0958, ZMO0961, ZMO1253* and *ZMO1255,* were reduced more than 1.5 times in ZM4 at pH 3.8 compared with pH 6.2 (Fig. 5f; Additional file 3: Table S2). In addition, six genes encoding Rnf complex (*ZMO1809–ZMO1814*) and an assembly gene (*ZMO1808*) were also downregulated at pH 3.8 compared with pH 6.2 in ZM4 but not in mutant strains (Fig. 5f; Additional file 3: Table S2). The Rnf complex is required for the electron transfer to nitrogenase during nitrogen fixation with proton excretion in *Rhodobacter capsulatus* [80]. Furthermore, the gene *ZMO0456* encoding the ferredoxin, which is the electron acceptor from NADH and electron donor for nitrogenase, was also downregulated at acidic pH 3.8 compared with neutral pH 6.2 in ZM4 (Fig. 5f; Additional file 3: Table S2). The downregulation of genes associated with the electron transfer chain at the acidic-pH condition in wild-type ZM4 could make the excretion of protons against proton gradient from cytoplasm difficult, leading to growth inhibition. In contrast, the expression of these genes in the mutant background was not significantly downregulated at acidic pH 3.8 compared with neutral pH 6.2. Instead, they were upregulated compared with ZM4 at pH 3.8 (Fig. 5f; Additional file 3: Table S2). These results indicated that mutants could maintain relatively high proton transportation capacity against acidic-pH conditions.

#### Proton consumption and alkaline compound production for enhanced acidic-pH resistance

Biosynthesis of branched-chain amino acids (BCAAs) was reported to reduce H^+^ concentration in the cytoplasm by consuming proton or producing ammonia [64]. Two genes involved in the conversion of isoleucine from threonine in *Z. mobilis* (*ZMO0687* and *ZMO0115*) were significantly upregulated in mutants 3.5M and 3.6M compared with ZM4 at pH 3.8 (Fig. 5G; Additional file 3: Table S2).

In addition, gene *ZMO0296* encoding adenosine deaminase (Ada) to convert adenosine into inosine with ammonia production was significantly upregulated at pH 3.8 in 3.6M strains compared with ZM4 (Fig. 5g; Additional file 3: Table S2). The expression of *ZMO1207* gene encoding nitrilase (Nit, EC 3.5.5.1) that catalyzes the substrate containing cyano group to ammonia was also upregulated at pH 3.8 in mutant strain 3.6M compared with ZM4 (Fig. 5g; Additional file 3: Table S2). At acidic pH conditions, ammonia could react with protons to produce the ammonium ion [81], which indicated that mutant strain 3.6M possessed greater capacity than mutant strain 3.5M and ZM4 to neutralize the intracellular pH by proton-consuming and alkali-producing reactions resulting in enhanced acidic-pH resistance.

However, the cytoplasmic pH homeostasis is connected with the proton motive force (PMF), which consists of two components of a transmembrane pH gradient (ΔpH) and a transmembrane electrical potential (Δψ) maintaining intercellular negative relative to outside [81]. The production of NH_4_^+^ from NH_3_ and proton thus will result in excess intracellular positive charges while reducing the ΔpH, which could destroy the PMF and impair cellular functions. To balance the excess intracellular positive charges, exporting NH_3_ and NH_4_^+^ by an ammonium transporter would avoid excessive positive charges hyperpolarizing the cell membrane [81]. Our RNA-Seq results showed that the transcriptional level of ammonium transporter encoded by *ZMO0346* was upregulated significantly in both mutant strains compared with ZM4 (Fig. 5g; Additional file 3: Table S2), which may help transport NH_3_ and NH_4_^+^ outside the cell and ensure normal PMF function on the membrane. Moreover, it was reported that the conversion of CO_2_ to HCO_3_^−^ by carbonate anhydrase (CA) also contributed to acid–base equilibrium in *H. pylori* [21, 81]. It is interesting that the transcriptional level of *ZMO1133* encoding carbonate anhydrase was significantly upregulated at pH 3.8 compared to that at pH 6.2 in all strains (Fig. 5g; Additional file 3: Table S2). Since *Z. mobilis* can consume sugars and produce CO_2_ efficiently [82], CO_2_/HCO_3_^−^ could also be involved in keeping acid–base equilibrium at acidic-pH conditions.

#### Reduced energy consumption on macromolecular repair for enhanced acidic pH tolerance of mutant strains

Cell membrane, proteins, and DNA would be damaged when bacteria are cultured in acidic environments. To reduce the damage, the expression of repair and defense proteins such as DnaK, RecA, UvrA, IrrE, and AP endonuclease could be increased to protect the macromolecules from the damage [64, 75, 83]. Our results showed that the transcription level of *ZMO0660* (*dnaK*) together with its co-chaperone *ZMO1690* (*dnaJ*) as well as *ZMO1588* (*uvrA*) and its subunit *ZMO0362* (*uvrB*) were upregulated in ZM4 at acidic pH 3.8 than at neutral pH 6.2. Moreover, the expression level of Clp protease complex, *ZMO0405* (*clpA*), *ZMO0948* (*clpP*), *ZMO0949* (*clpX*) and *ZMO1424* (*clpB*) involved in protein remodeling and reactivation [64, 84], altered similarly as *ZMO0660* (Fig. 5h; Additional file 3: Table S2). These results demonstrated that it is necessary to enhance the expression of these proteins in order to protect DNA and protein from damage in acidic cytoplasm.

However, the expression level of these genes was down-regulated at pH 3.8 in mutants compared to ZM4, except for gene *recA,* which had no significant changes at different pH conditions in any strains. In addition, the transcriptional level of *ZMO1929*, which encodes GroEL protein and is important during adaptation to acid [64], was downregulated at pH 3.8 in mutant strains compared to ZM4 (Fig. 5h; Additional file 3: Table S2). The deficient in HtrA, a surface protease involved in the degradation of aberrant proteins, reduced the ability of the mutant strain to endure acidic conditions [85], which demonstrated that this protein is important for cells to defend acid conditions.

The phenomenon that the expression of macromolecular repair genes that are indispensable for acid resistance was upregulated at acidic pH only in wild-type ZM4 background indicated that a great demand on these proteins is needed for ZM4 to survive at acidic-pH conditions, while the downregulation of these genes in mutant backgrounds compared with ZM4 suggested that acidic-pH-tolerant mutants may acquire the capability to manage defense responses without triggering abrupt augmented macromolecular repair activities and thus conserve energy for cell growth instead.

### Genetic confirmation of genes associated with acidic-pH resistance in *Z. mobilis* ZM4

**Fig. 6:**
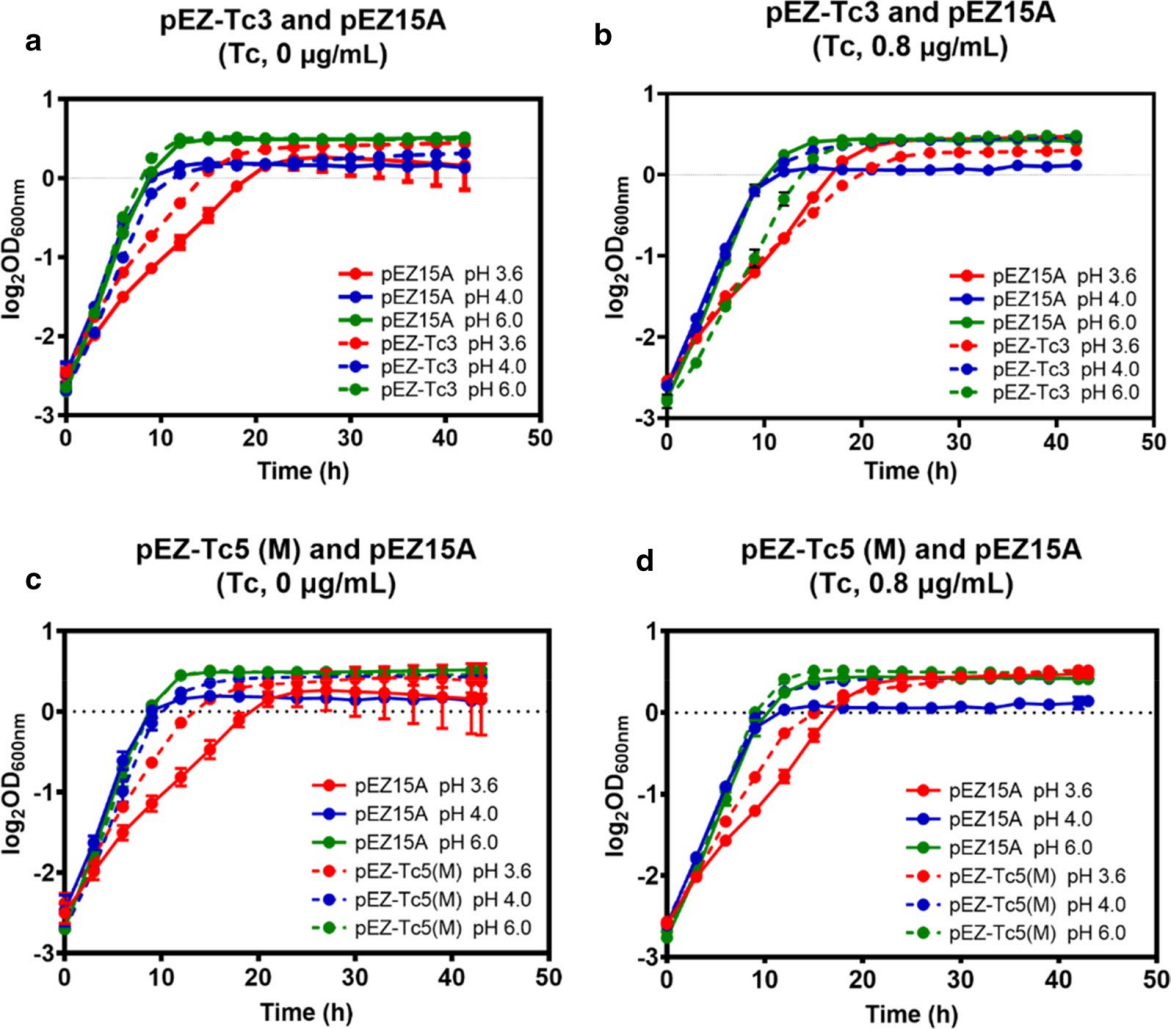
Cell growth of recombinant and wild-type strains of *Z. mobilis* containing the control plasmid pEZ15A and plasmid constructs of pET-Tc3 and pET-Tc5 (M) at pH 3.6, 4.0, and 6.0 without tetracycline (**a**, **c**) and with 0.8 μg/mL tetracycline induction (**b**, **d**). pEZ-Tc3, plasmid construct expressing operon *ZMO0956*–*ZMO0958* encoding cytochrome bc1 complex; pEZ-Tc5 (M), plasmid construct expressing operon *ZMO1428*–*ZMO1432* encoding RND efflux system with a mutation in *ZMO1432.* At least two independent experiments were performed with similar results. Values are the mean of one representative experiment with three technical replicates. Error bars represent standard deviations

To evaluate the impact of candidate genes associated with acidic-pH resistance identified through our genomic and transcriptomic studies as discussed above, six plasmids containing candidate operons were constructed based on the shuttle vector pEZ15Asp with P*tet* as the promoter [86]. These candidate operons including *ZMO0142–ZMO0145* encoding ABC transporter, *ZMO0798–ZMO0801* encoding multiple drug efflux, *ZMO0956–ZMO0958* encoding cytochrome bc1 complex, *ZMO0238*–*ZMO0242* encoding ATP synthesis F_1_ submits, *ZMO1428*–*ZMO1432* encoding RND efflux system with a mutation in *ZMO1432,* and *ZMO2005, ZMO0667*–*ZMO0671* encoding ATP synthesis F_0_ submits were cloned into pEZ15Asp shuttle vector, which were named pEZ-Tc1, pEZ-Tc2, pEZ-Tc3, pEZ-Tc4, pEZ-Tc5(M) and pEZ-Tc6, respectively. These plasmid constructs including the empty vector pEZ15Asp as the control were then introduced into ZM4 separately. These recombinant strains were then investigated under different conditions to examine their impact on cell growth (Fig. 6, Additional file 6: Fig. S4).

With the increase of the tetracycline inducer concentration from 0 to 0.8 μg/mL, the growth advantage of the recombinant strain containing pEZ-Tc3 decreased at pH 3.8 (Fig. 6a, b). Our previous work demonstrated that the P*tet* promoter driving the operon expression used in this study is leaky even when tetracycline was not supplemented into the medium [86, 87]. Therefore, this result suggested that the cytochrome bc1 complex encoded by the operon *ZMO0956–ZMO0958* in the recombinant strain containing pEZ-Tc3 could contribute to the acidic pH tolerance in *Z. mobilis* at a low expression level, which is consistent with our RNA-Seq result that the reduced expression of genes associated with electron transfer chain impacted the acid resistance of wild-type ZM4 (Fig. 5f; Additional file 3: Table S2). In addition, the upregulated expression of cytochrome bc1 complex impacted the growth in neutral pH condition (Fig. 6b), which may indicate a potential role of low expression of this “dead-end” respiration branch in cell growth, since the function of cytochrome bc1 in electron transport is still unknown in *Z. mobilis* [88]. Similarly, with the increase of tetracycline inducer concentration from 0 to 0.8 μg/mL, the growth advantage of recombinant strain containing pEZ-Tc5(M) decreased in acidic-pH condition, but there was still an advantage with 0.8 μg/mL tetracycline (Fig. 6c, d), which is again consistent with the upregulation of these genes in our RNA-Seq study (Fig. 5e; Additional file 3: Table S2). Although further investigation is still needed to understand the association of acidic-pH resistance with the different expression levels of these genes, our result suggested that the mutation in the intergenic region of upstream of *ZMO1432* in mutant 3.6M may contribute to the upregulation of the downstream gene, and a higher expression of RND efflux pump is more advantageous for strains to defend against acidic-pH conditions.

Recombinant strains containing the other four operons had no advantageous effect on cell growth in acidic-pH conditions both with and without tetracycline induction (Additional file 6: Fig. S4). Instead, the growth of the recombinant strain containing pEZ-Tc2 was dramatically impeded when induced with 0.8 μg/mL tetracycline (Additional file 6: Fig. S4C, S4D), and the growth of the recombinant strain containing pEZ-Tc6 was inhibited both with and without the supplementation of tetracycline inducer (Additional file 6: Fig. S4G, S4H). Since these operons encode the ABC transporter, multiple drug efflux, and ATP synthase submits, which may function with other cellular component coordinately, a delicate balance of these operons with other genes may be needed for acidic-pH resistance similar to a previous report’s findings that the tailored expression of multiple genes simultaneously was essential for enhanced low-pH tolerance in *E. coli* [89].

In summary, although our results suggested that the mutation in the intergenic region of upstream of *ZMO1432* in mutant 3.6M may contribute to the upregulation of the downstream gene (Table 2), and high expressions of RND efflux pump or cytochrome bc1 complex is advantageous for the strain to defend acidic-pH condition (Fig. 6), the advantage of these recombinant strains was still not as prominent as the mutant itself. This indicates that one gene/operon is not sufficient to warrant the acidic pH tolerance, and synergetic effects of multiple mutations affecting protein structural changes and expression of multiple genes need to be further investigated to understand the association of acidic-pH resistance phenotype with the genetic difference of mutant strains.

## Conclusion

Two acidic-pH-tolerant mutants 3.6M and 3.5M of *Z. mobilis*, which possessed advantages at acidic-pH conditions including high growth rate and ethanol productivity, were obtained from wild-type ZM4 by ALE strategy in this study. Genetic changes and gene expression at acidic and neutral pH conditions were then investigated using NGS-based genome resequencing and RNA-Seq with the underlying mechanism of acidic-pH resistance proposed. Specifically, *Z. mobilis* altered its metabolic flux through genomic changes affecting gene and gene expression associated with membrane modification, proton transportation, energy conservation and redistribution for acidic-pH resistance. Mutant strains had genes differentially expressed at acidic-pH conditions to help strengthen membrane-associated transporters, and increase proton consumption and alkaline metabolite production to maintain proton permeability and cellular pH homeostasis.

In addition, the enhanced F_1_F_o_ ATPase was also upregulated in mutant 3.6M, which could contribute to its advantage in the acidic-pH condition over another mutant 3.5M and wild-type ZM4. Genetic study results demonstrated that the introduction of plasmid constructs containing operons expressing cytochrome bc1 complex or RND efflux pump affected acidic pH tolerance in *Z. mobilis*. This study obtained and characterized acidic-pH resistant mutant strains of *Z. mobilis*, which can be used as candidate strains for commercial bioethanol production under acidic fermentation conditions. In addition, the molecular mechanism of acidic pH tolerance of *Z. mobilis* proposed in this study can also facilitate future research on rational design of synthetic microorganisms with enhanced tolerance against acidic-pH conditions, and the strategy we developed in this study combining ALE, genome resequencing, RNA-Seq, and classical genetic study for mutant evolution and characterization can be applied to other industrial microorganisms.

## Materials and methods

### Bacterial strain and culture conditions

*Escherichia coli* DH5α from Invitrogen (USA), was cultured in Lysogeny Broth (LB, 10 g/L NaCl, 10 g/L tryptone, 5 g/L yeast extract, and 1.5% agar for solid). *Z. mobilis* ZM4 (ATCC 31821) was cultured in Rich Medium (RM, 10 g/L yeast extract, 2 g/L KH_2_PO_4_, different concentration of glucose as required, and 1.5% agar for solid) at 30 °C with sharking at 100 rpm. The initial pH of culture medium was adjusted using HCl or KOH. For pH tolerance tests, glucose concentration in the media was 20.0 g/L (RMG2) and 50.0 g/L (RMG5); a flask containing RMG of its 80% volume (e.g., 400 mL RMG in a 500-mL flask) was used for shake-flask fermentation experiments.

### Adaptive laboratory evolution (ALE)

ALE was applied to evolve the wild-type strain *Z. mobilis* ZM4 (Fig. 2a). Cells were first streaked on RMG2 plate from stock; two colonies were then selected randomly and cultivated in liquid RMG2. After grown to the mid-exponential phase (OD_600_ = 0.80–2.50) as determined by a spectrophotometer UV-1800 (AOE, China), cells were then transferred into fresh RMG2 at pH 4.0 with the initial OD_600_ value of 0.1. When reaching to the mid-exponential phase, strains were transferred again into the same medium. After 30 cycles of cultivation at pH 4.0, the adapted strains were further transferred to fresh medium at pH 3.5 or pH 3.6. Finally, four evolved strains with enhanced tolerance to acidic pH were obtained, two adapted with 55 cycles of cultivation at pH 3.5 and the other two with 75 cycles of cultivation at pH 3.6 (Fig. 2a).

### Stability and pH tolerance evaluation of evolved cultures using Bioscreen C

Four evolved cultures were streaked on RMG2 plate, three colonies were then selected randomly and cultured overnight in 2 mL liquid RMG2 to the exponential phase. Seed cultures were washed twice and transferred into different media with an initial OD_600nm_ value of 0.1. Media were adjusted to pH 3.6 for the stability analysis or adjusted to pH 3.5, pH 3.6, pH 4.0, and pH 6.0, respectively, for the pH tolerance analysis. Cell growth was monitored at 600 nm using a Bioscreen C instrument (Growth Curves USA, NJ) with three technical replicates [90]. The working volume was 200 μL/well. The temperature was controlled at 30 °C and the absorbance values were automatically read at regular intervals of 15 min. The experiments were repeated at least twice.

### Cell growth and fermentation analysis

Fermentations were performed in 500-mL shake flasks with 400 mL RMG5 at 30 °C with an agitation rate of 100 rpm, and the wild-type ZM4 was used as the control. The pH of the media was adjusted at 3.8 and 6.2 by automatic titration with HCl and KOH. During the fermentation, Cell growth was determined every 2 h by measuring the optical density at 600 nm (OD_600_) using spectrophotometer (UV-1800, AOE, Chain). The samples were also collected and centrifuged at 10,000 rpm for 5 min, and filtered through 0.22-μm filters. Samples at the exponential phase were harvested for genome resequencing and RNA-Seq. Glucose and ethanol in filtered supernatants were analyzed using high-pressure liquid chromatography (HPLC, LC-20 AD, refractive index detector RID-10A, Shimadzu, Kyoto, Japan) with an Aminex HPX-87H column (Bio-Rad, Hercules, CA, USA) at 60 °C at the flow rate of 0.5 mL/min using 5 mM H_2_SO_4_ as the mobile phase.

### Whole-genome resequencing analysis

Whole-genome resequencing was performed using the paired-end sequencing technology according to standard Illumina protocols by IgeneCode, Inc., Beijing, China. The paired-end reads quality was checked using FastQC program (http://www.bioinformatics.babra ham.ac.uk/projects/fastqc/). Data passing the quality control were then mapped to the reference genome sequences of *Z. mobilis* ZM4, ATCC 31821 (GenBank accession No. of chromosome: NZ_CP023715, and plasmids: NZ_CP023716, NZ_CP023717, NZ_CP023718, NZ_CP023719) using the CLC Genomics Workbench (version 11.0) to identify the genomic variations. The objective mutations of the mutant strains were obtained with the parental wild-type strain as control, of which the mutation frequency more than 30% was filtered.

### RNA-Seq transcriptomic analysis

The transcriptomic study was carried out as reported previously [45, 91]. Briefly, cell cultures were collected at the mid-log phase under different pH conditions, followed by total RNA extraction using TRIzol reagent (Invitrogen, USA). rRNA within total RNA was depleted using Ribo-off rRNA Depletion Kit (Bacteria NR407). The fragmentation buffer was then added for interrupting mRNA to short fragments, and random hexamer-primers were used to synthesize the first-strand cDNA. The second-strand cDNA was synthesized using buffer, dATPs, dGTPs, dCTPs, dUTPs, RNase H and DNA polymerase I, respectively, after removing dNTPs. The fragments were connected with sequencing adapters for sequencing library construction, which was then sequenced using Illumina NovaSeq 6000 System.

Data passing the quality control were imported into CLC Genomics Workbench (version 11.0) for RNA-Seq analysis to get the RPKM values with *Z. mobilis* ZM4, ATCC 31821 (GenBank accession No. of chromosome: NZ_CP023715, and plasmids: NZ_CP023716, NZ_CP023717, NZ_CP023718, NZ_CP023719) and four related plasmids as the reference genome. Gene expression normalization, analysis of variance (ANOVA), and hierarchical clustering analysis were conducted using JMP Genomics (version 9.0) to identify differentially expressed genes at different conditions. Significantly differential expression genes were determined with a selection threshold of *P*-value ≤ 0.01 and log_2_-fold change ≥ 1 (significant induction) or ≤ −1 (significant repression). Duplicate samples were used for each condition.

### Genetic study evaluating the impact of candidate operons on acidic pH tolerance

Six operon candidates, *ZMO0142*–*ZMO0145*, *ZMO0798*–*ZMO0801, ZMO0956*–*ZMO0958, ZMO0238*–*ZMO0242, ZMO1429*–*ZMO1432, ZMO2005* with *ZMO0667*–*ZMO0671,* encoding ABC transporter related protein, multiple drug efflux, cytochrome bc1 complex, ATP synthesis F_1_ submits, RND efflux pump, ATP synthesis F_0_ submits, were amplified from the genomic DNA of *Z. mobilis* except that *ZMO1429*–*ZMO1432* was amplified from the genomic DNA of mutant strain with a mutation on the gene *ZM01432* using primers listed in Additional file 7: Table S3. The PCR products were then cloned into the pEZ15Asp shutter vector with P*tet* as the promoter [86] by the Gibson assembly method [92]. Each primer for operon amplification contained a 15 ~ 20 nucleotide overlapping region of the vector. Recombinant strains containing correct plasmid constructs were identified by colony PCR and confirmed by Sanger sequencing (Tsingke, China). These plasmids were named as pEZ-Tc1, pEZ-Tc2, pEZ-Tc3, pEZ-Tc4, pEZ-Tc5(M), and pEZ-Tc6, respectively.

The correct plasmids were then transformed into *Z. mobilis* competent cells via electroporation (0.1-cm electrode gap, 1600 V, 200 Ω, 25 μF) using a Gene Pulser^®^ (Bio-Rad, USA) as described previously [93]. Candidate strains containing correct plasmid construct were identified by colony PCR, and confirmed by Sanger sequencing (Tsingke, China). Cell growth of these strains was evaluated at different pHs (3.6, 4.0, 6.0) in RMG5 medium using Bioscreen C. Tetracycline was added at concentrations of 0.4 μg/mL and 0.8 μg/mL to induce genes expression.

## Supplementary information

**Additional file 1: Table S1.** List of all significantly differentially expressed genes between different strains in different conditions. *P* is −log10(*P*-value).

**Additional file 2: Fig. S1.** The Venn diagrams of significantly differentially expressed genes of same strain under different pH conditions **(A)** and two different strains at acidic pH condition **(B).**

**Additional file 3. Table S2.** List of significantly differentially expressed genes in different functional categories comparing same strain at different conditions or different strains under low pH. *P* is −log10(*P*-value). NS, not significantly differentially expressed.

**Additional file 4: Fig. S2.** The transmembrane domain prediction of the inner membrane component of RND efflux system without mutation (**A**) and with mutation (**B**) using TMHMM. The arrow points the T11 domain, where the mutation located.

**Additional file 5: Fig. S3.** Acetate production of *Z. mobilis* wild-type ZM4 and mutant strains of 3.5M and 3.6M at pH 3.8 and 6.2 when the glucose was completely consumed. At least two independent experiments were performed with similar results. Values are the mean of one representative experiment with three technical replicates. Error bars represent standard deviations. Statistics analysis was calculated using one-way ANOVA by GraphPad Prism 8.3.0. ** indicates adjusted *p*-value < 0.01.

**Additional file 6: Fig. S4.** Cell growth of recombination strains and wild type of *Z. mobilis* containing the control plasmid pEZ15A and plasmid constructs of pEZ-Tc1, pEZ-Tc2, pEZ-Tc4, and pEZ-Tc6, respectively, at pH 3.6, 4.0, 6.0 without tetracycline induction (**A, C, E, G**), or with the induction of 0.8 μg/mL tetracycline (**B, D, F, H**). In these graphs, red line represents pH 3.6, blue line represents pH 4.0, green line represents pH 6.0 (solid line represents pEZ15A and dotted line represents pEZ-Tc1, pEZ-Tc2, pEZ-Tc4 or pEZ-Tc6, respectively). **pEZ-Tc1**, plasmid construct expressing operon *ZMO0142*-*ZMO0145* encoding ABC transporter related protein; **pEZ-Tc2**, plasmid construct expressing operon *ZMO0798*-*ZMO0801* encoding multiple drug efflux related proteins; **pEZ-Tc4**, plasmid construct expressing operon *ZMO0238 *~ *ZMO0242* encoding ATP synthesis F_1_F_0_ submits; **pEZ-Tc6**, plasmid construct expressing operon *ZMO2005, ZMO0667*-*ZMO0671* encoding ATP synthesis F_1_F_0_ submits. Experiments have been repeated at least three times with similar result, and results from one experiment with three triplicate technical repeats were presented.

**Additional file 7: Table S3:** Primers used in this study.

## Data Availability

The authors declare that all data supporting the findings of this study are available within the paper and its Supplemental files or from the corresponding author on request. The raw data of genome resequencing and RNA-Seq were deposited into Sequence Read Archive (SRA) database with the BioProject accession numbers of PRJNA590883 and PRJNA644990, respectively.

## Abbreviations

ABC: ATP-binding cassette
ADA: Adenosine deaminase
ALE: Adaptive laboratory evolution
ANOVA: Analysis of variance
BCAAs: Branched-chain amino acids
CA: Carbonate dehydratase
CDS: Coding sequence
CFAs: Cyclopropane fatty acids
DBD: DNA-binding domain
DEGs: Expressed genes
ED pathway: Entner–Doudoroff pathway
EMP pathway: Embden–Meyerhof–Parnas pathway
GRAS: Generally regarded as safe
HPLC: High-pressure liquid chromatography
HTH: Helix–turn–helix
LB: Lysogeny broth
mARTP: Multi-round atmospheric and room temperature plasma
NGS: Next-generation sequencing
NIT: Nitrilase
NTG: Nitrosoguanidine
PCR: Polymerase chain reaction
PMF: Proton motive force
polyP: Polyphosphate
PBP: Proton-buffering peptide
RD: Regulatory domain
RM: Rich medium
RNA-Seq: RNA sequencing
RND: Resistance nodulation-cell division
ROS: Reactive oxygen species
SNPs: Single-nucleotide polymorphisms
SNVs: Single-nucleotide variants
TM: Transmembrane
UFAs: Unsaturated fatty acids
WGR: Whole-genome resequencing
ΔpH: pH gradient
Δψ: Transmembrane electrical potential
