# Supplementary material for: Development and characterization of acidic-pH-tolerant mutants of Zymomonas mobilis through adaptation and next-generation sequencing-based genome resequencing and RNA-Seq
Source: Biotechnol Biofuels. 2020 Aug 13;13:144. doi: 10.1186/s13068-020-01781-1 (PMC7427070; doi:10.1186/s13068-020-01781-1)
Supplement: Supplementary file 2 — Additional file 2: Fig. S1. The Venn diagrams of significantly differentially expressed genes of same strain under different pH conditions (A) and two different strains at acidic pH condition (B). [file 13068_2020_1781_MOESM2_ESM.docx]

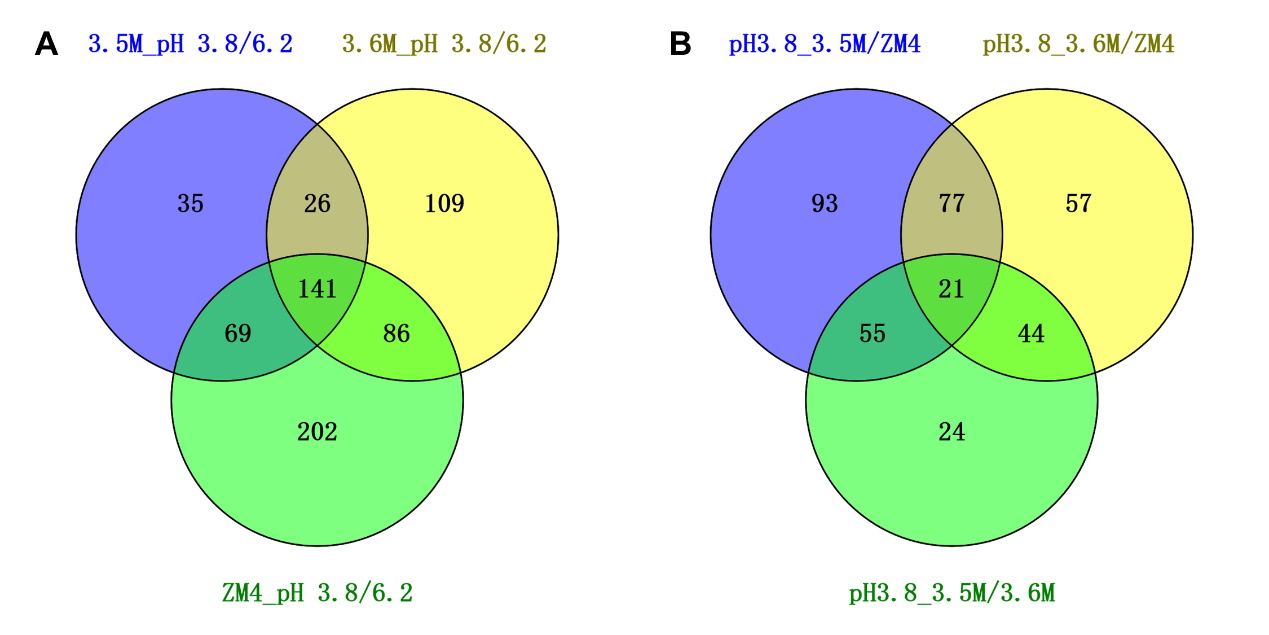


**Fig. S1.** The Venn diagrams of significantly differentially expressed genes of same strain under different pH conditions **(A)** and two different strains at acidic pH condition **(B)**.
