# Supplementary material for: Development and characterization of acidic-pH-tolerant mutants of Zymomonas mobilis through adaptation and next-generation sequencing-based genome resequencing and RNA-Seq
Source: Biotechnol Biofuels. 2020 Aug 13;13:144. doi: 10.1186/s13068-020-01781-1 (PMC7427070; doi:10.1186/s13068-020-01781-1)
Supplement: Supplementary file 4 — Additional file 4: Fig. S2. The transmembrane domain prediction of the inner membrane component of RND efflux system without mutation (A) and with mutation (B) using TMHMM. The arrow points the T11 domain, where the mutation located. [file 13068_2020_1781_MOESM4_ESM.docx]

**Fig. S2.** The transmembrane domain prediction of the inner membrane component of RND efflux system without mutation (**A**) and with mutation (**B**) using TMHMM. The arrow points the T11 domain, where the mutation located.
