# Supplementary material for: Development and characterization of acidic-pH-tolerant mutants of Zymomonas mobilis through adaptation and next-generation sequencing-based genome resequencing and RNA-Seq
Source: Biotechnol Biofuels. 2020 Aug 13;13:144. doi: 10.1186/s13068-020-01781-1 (PMC7427070; doi:10.1186/s13068-020-01781-1)
Supplement: Supplementary file 5 — Additional file 5: Fig. S3. Acetate production of Z. mobilis wild-type ZM4 and mutant strains of 3.5M and 3.6M at pH 3.8 and 6.2 when the glucose was completely consumed. At least two independent experiments were performed with similar results. Values are the mean of one representative experiment with three technical replicates. Error bars represent standard deviations. Statistics analysis was calculated using one-way ANOVA by GraphPad Prism 8.3.0. ** indicates adjusted p-value < 0.01. [file 13068_2020_1781_MOESM5_ESM.docx]

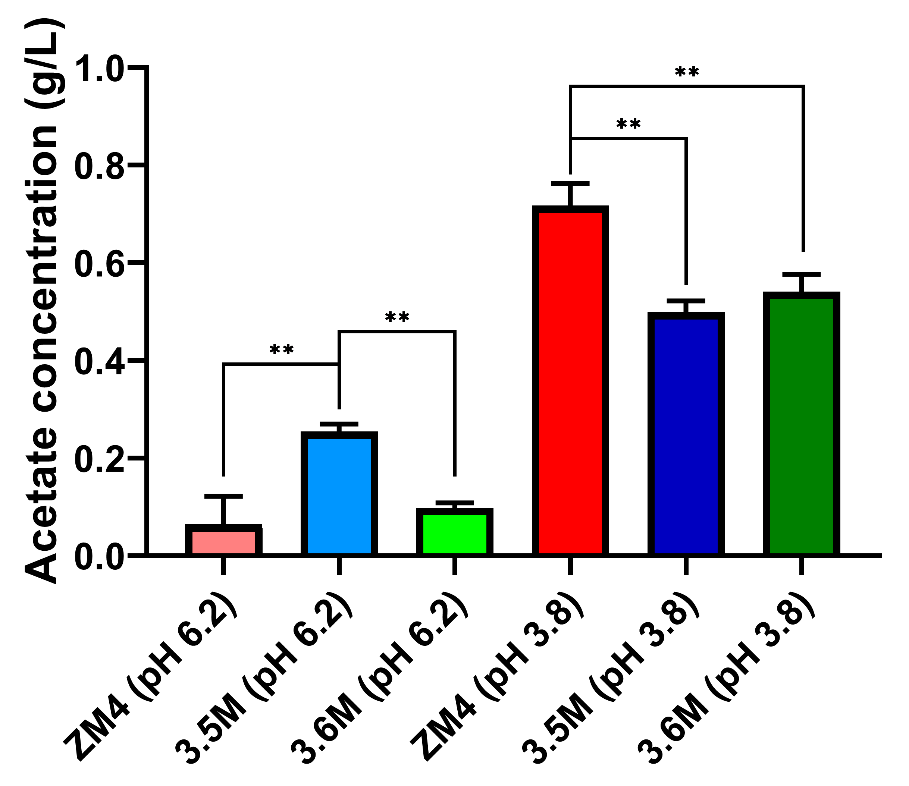


**Fig. S3.** Acetate production of *Z. mobilis* wild-type ZM4 and mutant strains of 3.5M and 3.6M at pH 3.8 and 6.2 when the glucose was completely consumed. At least two independent experiments were performed with similar results. Values are the mean of one representative experiment with three technical replicates. Error bars represent standard deviations. Statistics analysis was calculated using one-way ANOVA by GraphPad Prism 8.3.0. ** indicates adjusted *p*-value < 0.01.
