# Supplementary material for: Development and characterization of acidic-pH-tolerant mutants of Zymomonas mobilis through adaptation and next-generation sequencing-based genome resequencing and RNA-Seq
Source: Biotechnol Biofuels. 2020 Aug 13;13:144. doi: 10.1186/s13068-020-01781-1 (PMC7427070; doi:10.1186/s13068-020-01781-1)
Supplement: Supplementary file 6 — Additional file 6: Fig. S4. Cell growth of recombination strains and wild type of Z. mobilis containing the control plasmid pEZ15A and plasmid constructs of pEZ-Tc1, pEZ-Tc2, pEZ-Tc4, and pEZ-Tc6, respectively, at pH 3.6, 4.0, 6.0 without tetracycline induction (A, C, E, G), or with the induction of 0.8 μg/mL tetracycline (B, D, F, H). In these graphs, red line represents pH 3.6, blue line represents pH 4.0, green line represents pH 6.0 (solid line represents pEZ15A and dotted line represents pEZ-Tc1, pEZ-Tc2, pEZ-Tc4 or pEZ-Tc6, respectively). pEZ-Tc1, plasmid construct expressing operon ZMO0142-ZMO0145 encoding ABC transporter related protein; pEZ-Tc2, plasmid construct expressing operon ZMO0798-ZMO0801 encoding multiple drug efflux related proteins; pEZ-Tc4, plasmid construct expressing operon ZMO0238 ~ ZMO0242 encoding ATP synthesis F1F0 submits; pEZ-Tc6, plasmid construct expressing operon ZMO2005, ZMO0667-ZMO0671 encoding ATP synthesis F1F0 submits. Experiments have been repeated at least three times with similar result, and results from one experiment with three triplicate technical repeats were presented. [file 13068_2020_1781_MOESM6_ESM.docx]

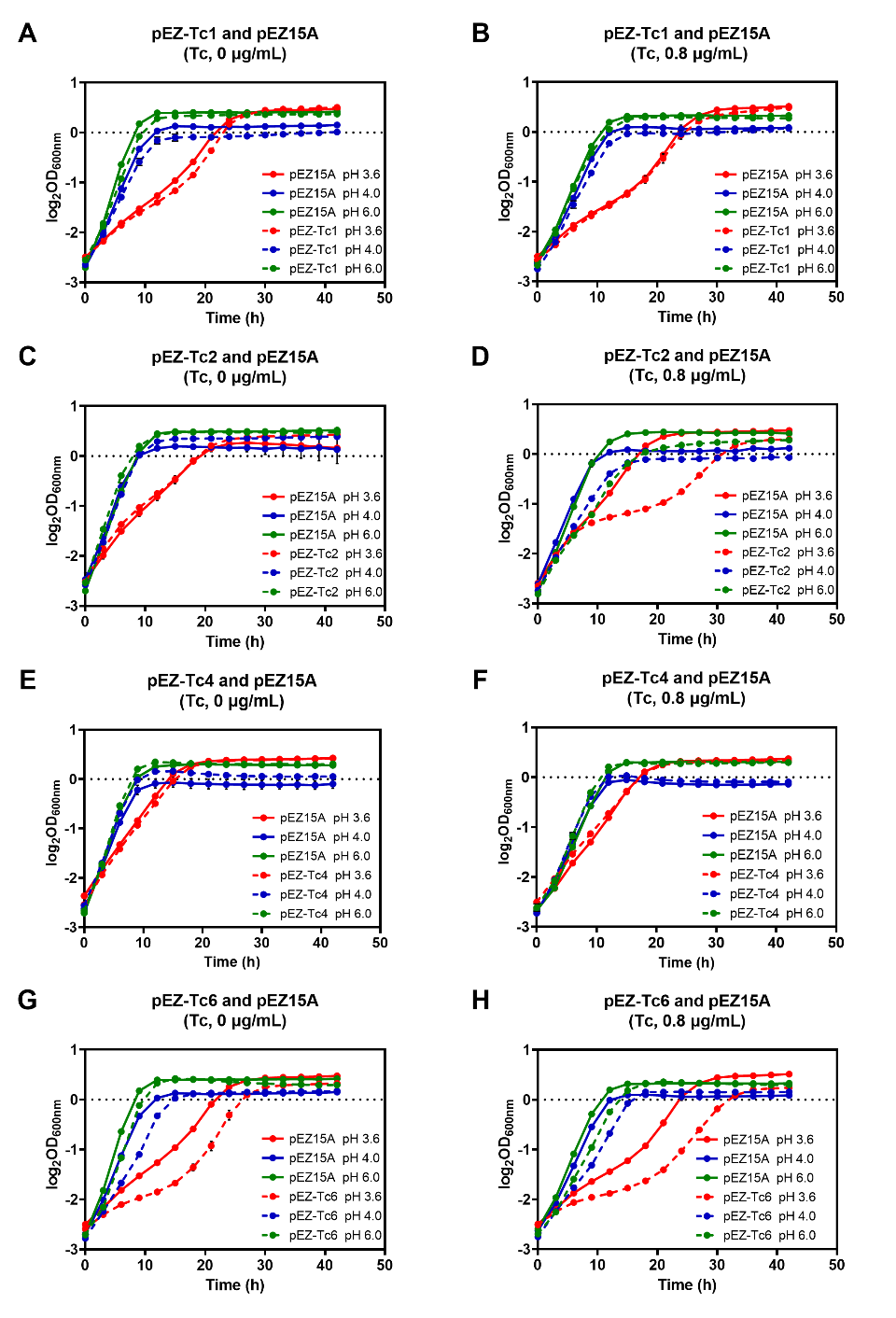


**Fig. S4.** Cell growth of recombination strains and wild type of *Z. mobilis* containing the control plasmid pEZ15A and plasmid constructs of pEZ-Tc1, pEZ-Tc2, pEZ-Tc4, and pEZ-Tc6, respectively at pH 3.6, 4.0, 6.0 without tetracycline induction (**A, C, E, G**), or with the induction of 0.8 μg/mL tetracycline (**B, D, F, H**). In these graphs, red line represents pH 3.6, blue line represents pH 4.0, green line represents pH 6.0 (solid line represents pEZ15A and dotted line represents pEZ-Tc1, pEZ-Tc2, pEZ-Tc4 or pEZ-Tc6, respectively). **pEZ-Tc1**, plasmid construct expressing operon *ZMO0142-ZMO0145* encoding ABC transporter related protein; **pEZ-Tc2**, plasmid construct expressing operon *ZMO0798-ZMO0801* encoding multiple drug efflux related proteins; **pEZ-Tc4**, plasmid construct expressing operon *ZMO0238~ZMO0242* encoding ATP synthesis F_1_F_0_ submits; **pEZ-Tc6**, plasmid construct expressing operon *ZMO2005, ZMO0667-ZMO0671* encoding ATP synthesis F_1_F_0_ submits*.* Experiments have been repeated at least three times with similar result, and results from one experiment with three triplicate technical repeats was presented.
