# Supplementary material for: Development and characterization of acidic-pH-tolerant mutants of Zymomonas mobilis through adaptation and next-generation sequencing-based genome resequencing and RNA-Seq
Source: Biotechnol Biofuels. 2020 Aug 13;13:144. doi: 10.1186/s13068-020-01781-1 (PMC7427070; doi:10.1186/s13068-020-01781-1)
Supplement: Supplementary file 7 — Additional file 7: Table S3: Primers used in this study. [file 13068_2020_1781_MOESM7_ESM.docx]

| **Table S3:** Primers used in this study. | |
| --- | --- |
|  |  |
| **Name** | **Sequence (5' to 3')** |
| Ptet-ZMO0142-F | aggagaaaggatctcccatgatagcgtcttctgccatgac |
| Terminator-ZMO0145-R | ctgcagcggccgctactagtttattctttggtgacagttttgg |
| Ptet-ZMO0238-F | aggagaaaggatctcccatggaaatatccggcagcattcaagc |
| Terminator-ZMO0242-R | ctgcagcggccgctactagtttagttttcttctttggcttcttctgc |
| Ptet-ZMO0801-F | aggagaaaggatctcccatgactgaccagacaaaagacgcc |
| Terminator-ZMO0798-R | ctgcagcggccgctactagtttagctccatccaccaccaag |
| Ptet-ZMO0956-F | aggagaaaggatctcccatgtcgaaagatgacgaaggtcactc |
| Terminator-ZMO0958-R | ctgcagcggccgctactagttcacaacttccgccatatccg |
| Ptet-ZMO1432-F | aggagaaaggatctcccatgctgtttaatttacggcaggcag |
| Terminator-ZMO1429-R | ctgcagcggccgctactagtttattcttttggtctttccaattcgc |
| Ptet-ZMO2005-F | aggagaaaggatctcccatgatgagtaacgattctaataaagggctg |
| Terminator-ZMO0671-R | ctgcagcggccgctactagttcagttaaggcgtgtgccttg |
| Ptet-F | catgggagatcctttctcctc |
| BioBrick-F | actagtagcggccgctgc |
